# Small Retailers' Tobacco Sales and Profit Margins in Two Disadvantaged Areas of England

**DOI:** 10.3934/publichealth.2016.1.110

**Published:** 2016-03-14

**Authors:** Sara C. Hitchman, Robert Calder, Catriona Rooke, Ann McNeill

**Affiliations:** 1. Department of Addictions, Institute of Psychiatry, Psychology, and Neuroscience, London, England; 2. UK Centre for Tobacco and Alcohol Studies, United Kingdom

**Keywords:** tobacco, tobacco retailers, tobacco economics, tobacco industry

## Abstract

**Aim:**

To explore tobacco profit margins and sales among small retailers in England.

**Methods:**

Interviews with managers/owners of 62 small retail shops that sold tobacco in disadvantaged areas of Newcastle and London, England. The interviews included questions about tobacco sales and profit margins, and interest in reducing reliance on tobacco sales.

**Results:**

The majority of retailers (89%) reported low overall profit margins on tobacco sales (< 6%). The most common response was a profit margin of 4–6%,with some reporting lower margins for price-marked packs of cigarettes (1–6%) and higher margins for non-price marked or premium brands (7% to over 10%). A few mentioned higher profit margins for e-cigarettes. Despite this, most thought tobacco sales were important (90%), and attributed this reliance to footfall (81%), i.e., customers purchasing tobacco also purchasing other products. 42% of retailers expressed interest in reducing their reliance on tobacco sales.

**Conclusions:**

Small retailers report low tobacco profit margins, but high reliance on tobacco sales because of footfall. Retailer interest in reducing reliance on tobacco sales warrants further research into opportunities for disinvestment. Additionally, retailers' belief that they are reliant on tobacco sales because of footfall should be further investigated.

## 1. Introduction

Previous research suggests the tobacco industry has remained profitable in the United Kingdom (UK) by utilising strategies such as market segmentation, thereby widening the gap between high (e.g., premium brand cigarettes) and low priced tobacco products and encouraging some smokers to down-trade to cheaper products rather than quitting [1],[2]. Less is known about small retailers' profits from tobacco and the extent to which they rely on tobacco sales. Several previous studies have explored the views of tobacco retailers in the UK regarding communication with the tobacco industry, tobacco advertising and pricing at the point of sale, and the display of e-cigarettes [3]–[6]. However none have examined small retailers' profit margins from tobacco and reliance on tobacco sales. To address this gap, we asked managers/owners of small retail shops in England about their tobacco sales and profit margins.

It is important to fill this gap in the research and improve our understanding of small retailers' stake in tobacco because retailers play an influential role in tobacco control policy debates. Small retailers made up the majority of business respondents who submitted responses to the UK Department of Health's consultation on standardised packaging, having sent a total of 10,001 ‘No to Plain Packs’ campaign postcards[7]. Additionally, the tobacco industry has used the importance of tobacco to retailers' livelihoods as an argument against standardised packaging[8]. Moreover, the continued availability of tobacco in small shops means that tobacco will remain a ubiquitous and readily accessible product for the time being. A greater understanding of retailer tobacco sales and profit margins will also aid with implementing Article 15 of the World Health Organization's Framework Convention on Tobacco Control [9]where signatories are required to regulate the production and distribution of tobacco products across the supply chain, including consideration of licensing of tobacco retailers.

## 2. Methods

The study was conducted in Newcastle upon Tyne (Newcastle) in North East England and London in South East England between August and October 2014. Wards (population of approx. 100,000) with predominantly disadvantaged populations were selected (3 in Newcastle and 5 in London). All small shops selling tobacco in the wards were identified and audited (n = 134) using methods from previous studies [5]. Small shops were defined as any shop under 280 sq. m/3,000 sq. ft. (this could be easily ascertained because at the time of the study these shops were still exempt from the ban on displaying tobacco at the point of sale in England which had been partially implemented for larger retailers in April 2012 and fully implemented (by small retailers) on 6 April 2015) [7]. Small shops were mapped by walking through streets one-by-one and visiting each shop; additional online checks (yell.com and www.192.com) were used to find shops that may have been missed. The audit consisted of recording the location of tobacco and any nicotine products, prominent brands, and anything else that stood out in the tobacco display (e.g., large price signs). Following each audit, researchers asked if the owner/manager would participate in an interview regarding tobacco issues, all who agreed were asked for consent and a time for the interview was scheduled with all retailers who consented. All shops were visited at least once to try and get an interview. The aim was to conduct 25–50 interviews in each city. It was decided to stop interviewing when data saturation was achieved and because of the time consuming nature of securing an interview with the shop manager/owner. In total 62 interviews were conducted, 32 in Newcastle and 30 in London. Each interview lasted approximately 20 minutes. This article focuses on the interviews, specifically questions related to tobacco sales and profit margins. The interview was structured and included closed and open ended questions, although the majority were open ended. At the end of the interviews retailers were given a chance to add additional comments. The interviewer noted responses during the interview. Open ended responses were categorized to simplify the presentation of results for this article. For further details on methods and other results see our previous report [10]. This study was approved by the Psychiatry, Nursing and Midwifery Research Ethics Committee at King's College London.

**Table 1.** Interviews with Managers and Owners of Small Shops: Profit Margins and Sales from Tobacco (N = 62).

Question	N	%
**Reliance on Tobacco Sales (N = 62)**		
Very important	27	43.5
Important	29	46.8
Not Important	3	4.8
Don't know/no opinion	3	4.8
**Reliance on tobacco sales due to footfall (N=62)**		
Yes	50	80.6
No	12	19.4
**Interest in reducing reliance on tobacco sales (N=62)**		
Yes	26	41.9
No	36	58.1
**Changes to tobacco sales (N=62)**		
Decrease	16	25.8
No change/mix of product change	35	56.5
Increase	3	4.8
Don't know	8	12.9
**Comments on change in mix of tobacco/nicotine product sales (N=28)**		
Decrease in premium/increase in cheaper brands and price-marked	14	50.0
Increase in smaller pack sizes (e.g., 19 pack instead of 20)	8	28.6
Increase in rolling tobacco sales	3	10.7
Increase in e-cigarette sales	3	10.7
Profit margins from tobacco sales (N=62)*		
**Overall profit margins (n=38)**		
1–3%	4	10.5
4–6%	30	78.9
7–10%	4	10.5
> 10%	0	0.0
**Profit margins from price marked tobacco (n=13)**		
1–3%	5	38.5
4–6%	8	61.5
7–10%	0	0.0
> 10%	0	0.0
**Profit margins from non-price marked or premium (n=10)**		
1–3%	0	0.0
4–6%	1	10.0
7–10%	7	70.0
> 10%	2	20.0

*Retailers quoted profit margins in different ways, some overall, and some by price-marked vs. non-price marked, percent out of those reporting for each category

## 3. Results

Table 1 shows the results. When asked how reliant they were on tobacco sales, the majority of retailers said tobacco sales were very important (43.5%) or important (46.8%). When asked if tobacco sales were important because tobacco attracts customers into the shop who may then purchase other products (i.e. “footfall”), 80.6% agreed [11]. When asked whether they were interested in reducing their reliance on tobacco sales, 41.9% either responded yes, or said yes, but qualified their response by saying they did not think it would be possible or they were not sure how they could reduce their reliance. When asked whether they had noticed any changes in tobacco sales recently, the majority (56.5%) reported no change or a change in the product mix. Nearly half (n = 28, 45.2%) mentioned a change in brands or the tobacco product mix, most of whom (n = 14, 50.0%) reported a decrease in premium brands and an increase in cheaper brands or price-marked cigarettes. Price marked cigarettes tend to be cheaper and carry a bold coloured price on the pack placed by the cigarette manufacturer, encouraging the retailer to selling it at a specified price (some retailers referred to them as ‘price locked.’). Retailers also noted an increase in smaller packs and a decrease in larger packs (e.g., 19 instead of 20 cigarettes, smaller pouches of rolling tobacco), increases in sales of rolling tobacco, and increases in sales of e-cigarettes.

When asked how much ‘profit’ they made from tobacco sales, most quoted an overall figure, with the vast majority of retailers reporting < 6%, with the majority saying 4–6%. A few retailers differentiated between price-marked and non-price marked or premium cigarettes. For price marked, the majority of retailers said they made a profit margin of 4–6%, but a large proportion also said they made 1–3%. For non-price marked or premium, the majority said they made a profit margin of 7–10%. A minority mentioned higher profit margins from e-cigarettes (n = 3).

## 4. Discussion

This study explored retailers' views on sales and profit margins from tobacco products within disadvantaged wards of Newcastle and London. Overall the findings suggest that despite reporting low profit margins, small shop owners/managers believe that tobacco is important for their business. A major reason that retailers remain reliant on tobacco is the perception that tobacco attracts customers into the shop who may then buy other products. A large minority of retailers expressed interest in reducing their reliance on tobacco. Most retailers reported no changes in tobacco sales, but instead changes in the mix of tobacco products sold, especially an increase in sales of cheaper tobacco products (e.g., price-marked, smaller pack sizes).

Retailers' perceptions that tobacco customers bring footfall is likely promoted by retailer trade magazine articles/advertisements. In a previous review [10] we noted that articles/advertisements in these magazines state that tobacco purchases bring footfall into shops and that tobacco customers spend more than other customers (e.g.,Imperial Tobacco, 2014; Japan Tobacco International, 2014b). However, because these articles/advertisements are often linked to the tobacco industry, it is uncertain if these statements are true. Because footfall is a key reason retailers believe they are reliant on tobacco sales, the statement that tobacco brings footfall should be further investigated.

Moreover, because some retailers have an interest in reducing their reliance on tobacco, future research should investigate how small retailers could disinvest from tobacco while at the same time maintaining profitability. This could be similar to studies exploring how tobacco farmers could switch to alternative crops with the goal of decreasing the supply of tobacco, a primary goal of the WHO's FCTC [9],[12]. Indeed, one retailer we spoke with was already in the process of disinvesting in tobacco and converting into a consumer goods shop. Disinvestment or reduced reliance on tobacco could also be worked into public health strategies that target small retailers or take the form of a new initiative, such as “Shop Healthy NYC,” which seeks to increase the supply and demand of healthy foods in small shops in New York City for the benefit of retailers and their communities, and, is promoted as a business opportunity for retailers[13].

In comparison to other products sold in small retail shops, profit margins from tobacco are low (4–6%). For example, profits margins on confectionary sales are estimated at 30%. Three retailers also mentioned higher profit margins from e-cigarettes. Advertisements in the retail trade press from non-tobacco industry e-cigarette companies also state higher profit margins, for example, one advertisement compares the profit margin of an e-cigarette (single disposable cigalike) at 40% to a premium pack of cigarettes at 6% [14],[15].

The higher profit margins quoted by retailers for non-price marked or premium tobacco brands reflects the higher sale prices and the higher profits made by the tobacco industry on premium brands[16]. Previous research suggests that the tobacco industry over-shifts cigarette tax increases to premium brands, keeping the price of cheaper cigarette brands low [16]. It is possible, as profits decrease on cheaper cigarettes for retailers that statements about the benefits of footfall will have to be strengthened or other strategies will be used to continue to convince retailers to sell cigarettes. Indeed, in our previous review of the retail trade literature[10], a closer look found instances where footfall from price marked and low price/value tobacco was emphasized [17],[18]. Unlike the tobacco industry, small retailers, particularly in disadvantaged areas, may not be able to make up for low profit margins on cheaper cigarettes if they do not sell enough premium brands. This merits further exploration.

Limitations of our study include that our sample, predominantly from disadvantaged areas, is not representative of all shops and it is possible that perceptions of profit margins differ in more advantaged areas. Previous research has indeed found that shops in disadvantaged areas tend to carry cigarettes with a lower average price [19]. Nevertheless, we interviewed a relatively large sample of small retailers in two distinct areas of England and our focus on disadvantaged areas was to reflect higher smoking prevalence in these areas. We also focussed on securing an interview with the shop manager or owner to ensure the information about profit margins and sales was as accurate as possible, even if this required several visits or making an appointment. Additionally, because this study was exploratory, the response options were open-ended and were categorised by the researchers. The benefit was that this led to insights for future research that would not have emerged in a forced response option questionnaire.

Funding: This research was funded by Cancer Research UK (Assessment of small retailer preparations for tobacco point of sale display removal and longer-term tobacco disinvestment). SCH and AM are part of the UK Centre for Tobacco and Alcohol Studies, a UK Clinical Research Collaboration Public Health Research Centre of Excellence. Funding from the Medical Research Council, British Heart Foundation, Cancer Research UK, Economic and Social Research Council and the National Institute for Health Research under the auspices of the UK Clinical Research Collaboration is gratefully acknowledged (MR/K023195/1).
